# Reply to: Response to limited surface impacts of the January 2021 sudden stratospheric warming

**DOI:** 10.1038/s41467-023-38773-2

**Published:** 2023-06-07

**Authors:** Nicholas A. Davis, Jadwiga H. Richter, Anne A. Glanville, Jim Edwards, Emerson LaJoie

**Affiliations:** 1 Atmospheric Chemistry Observations and Modeling Laboratory, National Center for Atmospheric Research, Boulder, CO USA; 2 Climate and Global Dynamics Laboratory, National Center for Atmospheric Research, Boulder, CO USA; 3 NOAA/NCEP/Climate Prediction Center, College Park, MD USA

**Keywords:** Atmospheric dynamics, Environmental impact

**replying to** J. Cohen et al. *Nature Communications* 10.1038/s41467-023-38772-3 (2023)

This response addresses concerns expressed in the Matters Arising, Cohen et al.^1^, published in response to Davis et al.^2^ Here, we will show that CESM2(WACCM6) is able to reproduce the stratospheric wave reflection and stratospheric wave forcing argued^1,3^ to be paramount to the February 2021 cold air outbreak, contrary to the claims in Cohen et al.^1^ We agree that the model does not reproduce the full severity of the cold air outbreak^1^, potentially due to the inability of the model to fully capture the strength of wave activity flux (WAF) convergence within the trough. However, we will show the strength of this WAF convergence is unrelated to the stratospheric wave dynamics, which supports our conclusion^2^ that polar vortex stretching and wave reflection did not play a measurable role in the cold air outbreak.

**Fig. 1:**
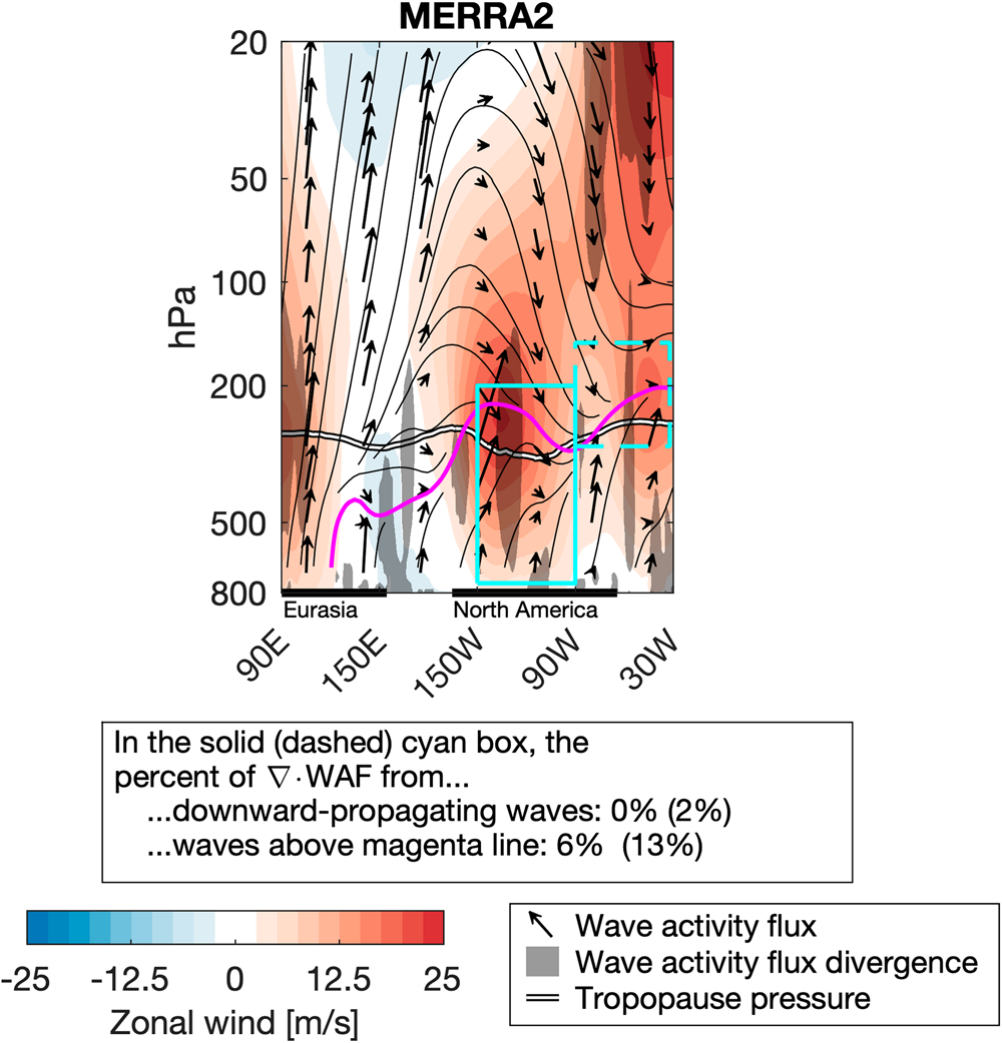
Midlatitude wave dynamics from February 1-12, 2021, preceding the cold air outbreak. Reproduced Fig. 7a from Davis et al.^2^ with the addition of several cyan boxes indicating averaging regions, as well as a magenta-highlighted wave activity flux (WAF) streamline. The contribution of reflected WAF to the integrated WAF convergence in the cyan boxes is indicated in the panel. Fields averaged from 45–75 N from February 1-12, 2021.

Figure 1 displays the midlatitude wave dynamics preceding the February 2021 cold air outbreak. Wave activity flux (WAF) generated over Eurasia propagated upward into the stratosphere, where it was reflected downward and eastward toward the troposphere, while waves generated at the surface over the Pacific Ocean and North America propagated upward to the tropopause layer, where they turned eastward and converged into the trough.

The question is how much the WAF reflected off the stretched stratospheric polar vortex contributed to the cold air outbreak. We agree that WAF convergence is a useful metric for the direct impact of the reflected waves^1^, though there is some disagreement over the precise region of interest. For instance, Cohen et al.^1^ note the WAF forcing downstream of the trough in the stratosphere may be important, and that we need to expand the dates we analyze up to the day before the event.

We can quantify the direct contribution of reflected waves to the wave forcing over two different regions of interest, indicated by the cyan boxes in Fig. 1, using two different diagnostics and over an expanded date range. In the first diagnostic, we integrate the WAF convergence in each cyan box wherever there is downward-oriented WAF (in other words, potentially reflected WAF), and compare it to the total integrated WAF convergence over each cyan box. This diagnostic is intended to capture all reflected waves - those reflected off the polar vortex, but also those reflected off the tropopause inversion layer, or just otherwise traveling downward. For the second diagnostic, we integrate *all* WAF convergence above the magenta streamline in each cyan box, and compare it to the total integrated WAF convergence over each cyan box. The magenta streamline was automatically generated by the plotting routine used to generate Fig. 7 from Davis et al.^2^ and acts as a useful boundary to segregate waves reflected off the polar vortex from waves traveling along the tropopause. We integrate in meters.

In the trough, 0 to 6% of WAF convergence is associated with reflected WAF, while downstream of the trough, 2 to 13% of WAF convergence is associated with reflected WAF (Fig. 1). Our experimental results^2^ are consistent with this analysis of the observed wave dynamics, which shows that WAF reflected off the polar vortex was a negligible contribution to the WAF convergence within the trough, and a small contribution to the WAF convergence downstream of the trough. Non-reflected WAF traveling upward from the surface dominated the WAF convergence within and downstream of the trough^2^.

**Fig. 2:**
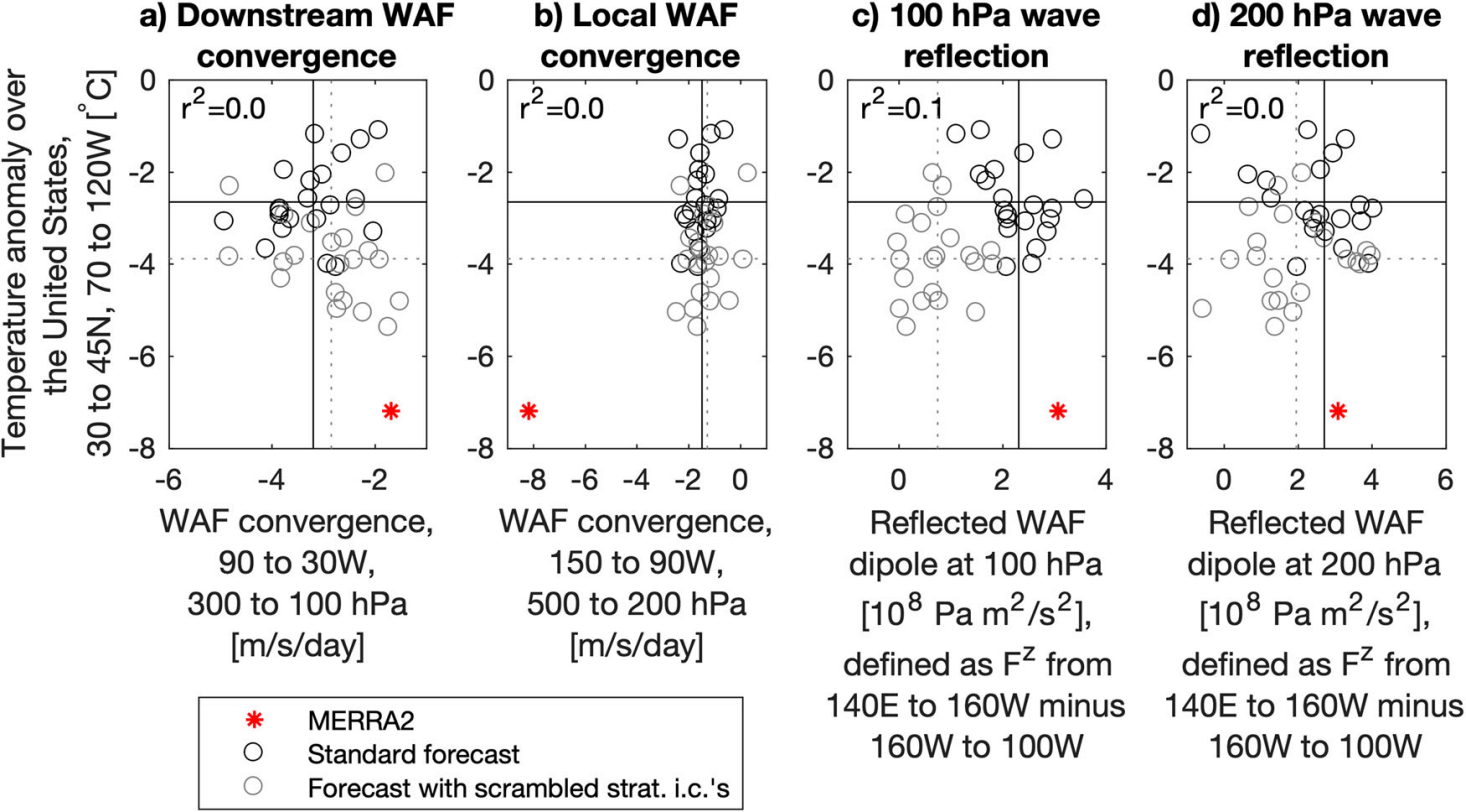
An assessment of CESM2(WACCM6) wave dynamics and their relationship with surface temperature anomalies during the cold air outbreak. Wave activity flux (WAF) convergence and WAF reflection in CESM2(WACCM6) and MERRA2 from February 1–12, 2021 versus the temperature anomaly over the United States from February 12–18, 2021, with the region defined approximately as in Cohen et al. (2023) from 30 to 45 N, 70 to 120 W. Wave dynamics shown are the (**a**) WAF convergence downstream of the trough in the stratosphere (dashed cyan region in Fig. 1), (**b**) WAF convergence within the trough region (solid cyan region in Fig. 1), (**c**) wave reflection at 100 hPa, and (**d**), wave reflection at 200 hPa. Solid black and dotted gray lines denote the mean values from the CESM2(WACCM6) ensembles. The correlation coefficient squared across CESM2(WACCM6) ensemble members is displayed in the upper left of each panel.

Even if it did not originate from WAF reflection off the vortex, WAF convergence downstream of the trough within the stratosphere may have played a role in the event. Here, we find CESM2(WACCM6) slightly over-predicts the WAF convergence in the stratosphere downstream of the trough compared to MERRA2 (Fig. 2a). There is no relationship between the WAF convergence in this region and the severity of the cold air outbreak.

Cohen et al.^1^ state that CESM2(WACCM6) does not reproduce the strength of local WAF convergence in the trough, which is indeed the case (Fig. 2b). If one considers MERRA2 and the two forecast ensembles as a combined sample, then there may be some relationship between the WAF convergence in the trough and the severity of the cold air outbreak, though it is not apparent within the model ensemble itself. Instead, one can find surface temperature anomalies ranging from −1 to −5 °C at a given value of WAF convergence.

Cohen et al.^1^ argue that wave reflection in the stratosphere is important in driving these events, and use 100 hPa to diagnose this process^3^. Here, we create a wave reflection index defined as the average vertical WAF from 140E to 160 W minus the average vertical WAF from 160 W to 100 W at 100 hPa, which when positive indicates strong upward WAF over Eurasia and strong downward WAF over North America. At 100 hPa, the standard forecast produces a strong dipole similar to what is observed in MERRA2 (Fig. 2c). Crucially, the forecast with scrambled stratospheric initial conditions exhibits virtually no wave reflection at this level. This demonstrates that our original experimental design^2^ was appropriate for testing the impact of wave reflection off a stretched polar vortex.

Cohen et al.^1^ suggest wave reflection in the lower stratosphere can influence cold air outbreaks. While this is not the mechanism we tested in Davis et al.^2^, we will examine it here by looking at the same dipole diagnostic at 200 hPa (Fig. 2d). The standard forecast reproduces the reflection observed in MERRA2, while the forecast with scrambled stratospheric initial conditions exhibits reflection slightly weaker than what was observed. Neither the tropospheric (Fig. 2b) nor lower-stratospheric (Fig. 2d) WAF fundamentally differ when wave reflection off the polar vortex is experimentally suppressed in the stratosphere (Fig. 2c), which suggests there is probably no “trapping” mechanism at work during this event, as suggested in Cohen et al.^1^

Across ensemble members, and between ensemble members and MERRA2, the strength of stratospheric wave reflection at both 200 and 100 hPa has no relationship with the severity of the cold air outbreak (Fig. 2c, d). Further, CESM2(WACCM6) simulated the full range of stratospheric wave dynamics (Fig. 2a, c, d). If there is any process that prevented CESM2(WACCM6) from simulating the full severity of the cold air outbreak, it is not the stratospheric wave dynamics, but the strength of WAF convergence in the trough (Fig. 2b), which is entirely dominated by upward-propagating WAF from below (Fig. 1) and unrelated to WAF at either 100 or 200 hPa (Fig. 2c, d).

Recent work suggests that localized tropospheric wave breaking in the Siberian and Labrador Seas^4^, as well as vertical mixing^5^ of radiatively-cooled surface air in Canada due to broad snow cover^6^, may have contributed to the severity of the cold air outbreak. CESM2(WACCM6) has 100 km horizontal resolution, with limited vertical resolution in the boundary layer. The model resolution may have been too coarse to capture these processes, and therefore underestimated the magnitude of the cold air outbreak in the ensemble mean.

Cohen et al.^1^ take issue with our experimental design, namely that the initialization tapers from full initialization below 9 km to none above 12 km. They state the 9–12 km layer “*includes the critical region for wave reflection near the tropopause and lower stratosphere*”. We think it’s important to note the distinction between wave reflection off the tropopause inversion layer and wave reflection off the polar vortex. Our initialization scheme fully tapers at 12 km, which is 5 km below the 100 hPa level commonly taken as the start of the stratospheric polar vortex.

The purpose of our study was to test whether wave reflection off the polar vortex influenced the cold air outbreak of February 2021^2^. Our purpose was not to test whether reflection off the tropopause inversion layer influenced the cold air outbreak. We believe arguing that our initialization “*cannot fully remove the influence of the lower stratosphere*”^1^ is a misdirection from the intent of our experiment, which was to remove the influence of wave reflection off the polar vortex.

As a remedy, Cohen et al.^1^ instead propose, as one example, to taper the initialization from 4 km to 7 km, which shifts the tested mechanism to reflection along and below the tropopause—which is not what we sought to test^2^ and not what we understood to be the proposed mechanism^3^. As our experimental design^2^ effectively shut down wave reflection off the polar vortex at the level^3^ used to diagnose reflection (Fig. 2c), tapering the initialization to zero by 12 km is an effective way of separating the influence of the anomalous polar vortex. Cohen et al.^1^ also claim that nudging the stratosphere through the duration of the event will provide a more accurate estimate of surface impacts. Our initial condition scrambling approach is an important contribution as it does not implicitly assume that the polar vortex is an external forcing on the troposphere, but instead that the two evolve as a coupled system. Initial condition scrambling is an experimental approach that can *uncouple* coupled systems.

Regarding the surface temperature anomaly predictions of the forecasts, Cohen et al.^1^ state the model predictions do not reproduce severe cold. Our subseasonal-to-seasonal forecast ensemble is purposely initialized with substantial spread, so the ensemble mean will not always capture an extreme event. Additionally, our analysis focused on the planetary-scale waves hypothesized^3^ to influence cold air outbreaks. We envisioned that taking a planetary-scale approach to the surface analysis^2^ would mesh better with the function of the ensemble and the scale of the hypothesized mechanism^3^, but we see that it has instead engendered the belief that this model could not reproduce the event at all.

**Fig. 3:**
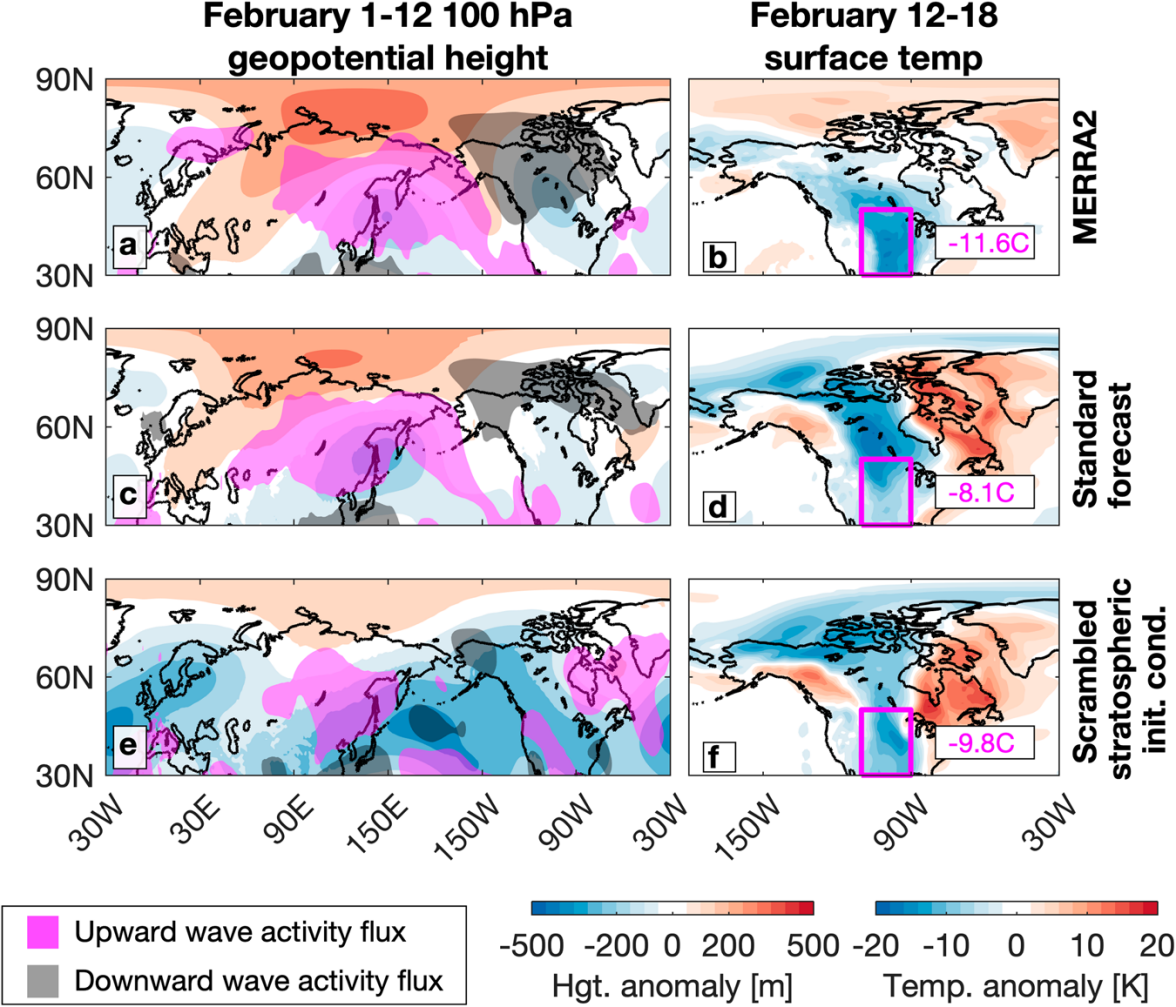
Stratospheric wave dynamics and surface temperatures over North America. **a**, **c**, **e** 100 hPa geopotential height anomalies and vertical wave activity flux as in Fig. 5 of Davis et al.^2^, with wave activity flux contour threshold halved, except here in (**c**) and (**e**) for the ensemble member initialized February 1 with the coldest surface temperature forecast in the magenta box. Surface temperature anomalies in (**b**) MERRA2 and (**d**, **f**) in each member initialized February 1 with the coldest surface temperature forecast in the magenta box.

There are ensemble members that predicted extreme cold over the Great Plains and Texas (Fig. 3d, f). A member from the standard forecast produced an average surface temperature anomaly of −8.1 °C compared to the observed −11.6 °C (Fig. 3b, d), and reproduced the observed wave reflection process at 100 hPa (Fig. 3a, c). On the other hand, a member with scrambled stratospheric initial conditions produced an anomaly of −9.8 °C (Fig. 3f) with no wave reflection and net upward wave propagation over North America (Fig. 3e). Given the same tropospheric initial conditions, whether one reproduces the full breadth of stratospheric wave dynamics (Fig. 2) or eliminates^2^ the key hypothesized mechanism^3^, our experiments^2^ show that wave reflection off the polar vortex had no tangible impact on the February 2021 cold air outbreak, within individual members (Fig. 3) and among the ensemble distribution (Fig. 4 in Davis et al.^2^).

Millin et al.^7^ show that there are two primary circulation regimes—the Arctic High and Alaskan Ridge - associated with cold air outbreaks in the Great Plains and Texas, with markedly different structures of variability. The February 2021 event resembles the Arctic High pattern, with elevated polar cap geopotential heights (compare Fig. 11 of Millin et al.^7^ to Fig. 3a of Davis et al.^2^), which is the regime that is generally associated with weak stratospheric wave reflection (Fig. 8 of Millin et al.^7^). On the other hand, there are cold air outbreaks associated with strong stratospheric wave reflection, but they tend to occur in an Alaskan Ridge regime^7,8^.

In sum, we believe CESM2(WACCM6) is useful for studying the connection between planetary-scale wave reflection off the polar vortex and cold air outbreaks. CESM2(WACCM6) simulated the observed stratospheric wave dynamics leading up to the event (Fig. 2), and while it did not capture the full strength of WAF convergence in the trough, this convergence was associated with upward propagating WAF from the surface. It does not appear to be associated with stratospheric wave dynamics, either directly (Fig. 1) or indirectly (Fig. 2). Our analysis of the event is also consistent with analyses of all cold air outbreaks in this region^7^.

There may be a connection between wave reflection off the polar vortex and some cold air outbreaks, but we feel the weight of the evidence suggests the February 2021 event was not one of them.
